# Alterations of Interhemispheric Functional Connectivity and Degree Centrality in Cervical Dystonia: A Resting-State fMRI Study

**DOI:** 10.1155/2019/7349894

**Published:** 2019-04-24

**Authors:** Wenyan Jiang, Yiwu Lei, Jing Wei, Lu Yang, Shubao Wei, Qiong Yin, Shuguang Luo, Wenbin Guo

**Affiliations:** ^1^Department of Neurology, The First Affiliated Hospital of Guangxi Medical University, Nanning, Guangxi 530021, China; ^2^Department of Radiology, The First Affiliated Hospital, Guangxi Medical University, Nanning, Guangxi 530021, China; ^3^Department of Psychiatry, The Second Xiangya Hospital of Central South University, Changsha, Hunan 410011, China

## Abstract

**Background:**

Cervical dystonia (CD) is a neurological movement disorder characterized by involuntary head and neck movements and postures. Reports on microstructural and functional abnormalities in multiple brain regions not limited to the basal ganglia have been increasing in patients with CD. However, the neural bases of CD are unclear. This study is aimed at identifying cerebral functional abnormalities in CD by using resting-state functional magnetic resonance imaging (rs-fMRI).

**Methods:**

Using rs-fMRI data, voxel-mirrored homotopic connectivity (VMHC) and degree centrality were used to compare the alterations of the rs-functional connectivity (FC) between 19 patients with CD and 21 healthy controls. Regions showing abnormal FCs from two measurements were the regions of interest for correlation analyses.

**Results:**

Compared with healthy controls, patients with CD exhibited significantly decreased VMHC in the supplementary motor area (SMA), precuneus (PCu)/postcentral gyrus, and superior medial prefrontal cortex (MPFC). Significantly increased degree centrality in the right PCu and decreased degree centrality in the right lentiform nucleus and left ventral MPFC were observed in the patient group compared with the control group. Further correlation analyses showed that the VMHC values in the SMA were negatively correlated with dystonia severity.

**Conclusion:**

Local abnormalities and interhemispheric interaction deficits in the sensorimotor network (SMA, postcentral gyrus, and PCu), default mode network (MPFC and PCu), and basal ganglia may be the key characteristics in the pathogenesis mechanism of CD.

## 1. Introduction

Cervical dystonia (CD) is the most common form of primary focal dystonia with adult onset; it is characterized by excessive involuntary contractions of the neck muscles, thereby leading to abnormal head movements and postures [1, 2]. CD often occurs in combination with head tremor or jerking movements [3]. The motor symptoms often accompany nonmotor symptoms, including sensory symptoms (pain), psychiatric disorders (depression and anxiety), sleep disorders, and cognitive disturbances [4]. This chronic disease affects functional ability and reduces work productivity, thereby resulting in high individual and social burden [5]. In most cases, CD is classified as primary because the etiology and pathophysiological mechanisms are unclear. Therefore, the disorder occurs for the rest of the patient's life without prolonged periods of spontaneous remission and curative therapy.

Three general abnormalities underlie the physiological substrate of dystonia, as follows: abnormal sensorimotor integration, loss of inhibition, and maladaptive neural plasticity [6]. However, the most plausible mechanism by which dystonia occurs and corresponding brain regions in the different forms of dystonia remain unclear. Although traditional pathophysiological dystonia models often are interpreted in the context of basal ganglia dysfunction [7–9], the contribution of many other brain regions has become obvious after considering evidence from various sources, including the modern neuroimaging [10–12]. Evident brain anatomical lesions are absent in the routine clinical imaging studies for individuals with primary dystonia. With the development of advanced magnetic resonance imaging technology, microstructural and functional abnormalities in multiple brain regions have been visualized, thereby extending the traditional focus. For example, some magnetic resonance imaging-based structural neuroimaging studies, involving the voxel-based morphometry and/or diffusion tensor imaging methods, revealed microstructural alterations in different brain areas, including the cortical regions of sensorimotor, temporal, parietal, and occipital areas, as well as the subcortical regions of the basal ganglia and cerebellum [13]. Functional neuroimaging techniques including positron emission tomography (PET) [14] and task-related functional magnetic resonance imaging (fMRI) [15] have detected abnormal functional architecture of the brain involving multiple regions. These imaging studies showed regional microstructural and functional abnormalities in multiple brain regions, thereby supporting the new concept that dystonia may be a network disorder, and regional abnormalities represent the changes in the network's nodes. Network disorder can arise from either dysfunction in several nodes or aberrant functional connectivity (FC) among the nodes.

Brain imaging techniques are noninvasive means in identifying clues to the understanding of pathophysiology of dystonia at the system level. Resting-state fMRI (rs-fMRI) has attracted increasing attention because it is an effective way to characterize intrinsic FC alterations in brain disorders [16], thereby offering extended insight into the underlying brain mechanisms. To date, few FC investigations on the basis of rs-fMRI data have been conducted on patients with CD [17–19]. An initial rs-fMRI study on CD demonstrated altered FC in the sensorimotor network (SMN), executive control network, and primary visual network via the independent component analysis (ICA) method [17]. Subsequently, the investigators utilized a seed-based correlation analysis and detected altered FC between the basal ganglia and some cortical regions within SMN and left frontoparietal network [18]. Another study showed that regional and interregional connectivity alterations are extensively distributed in the cortical and subcortical structures, and common alterations are identified bilaterally in the postcentral gyrus and in the basal ganglia and thalamus [19]. These studies provide support for the aberrant neural coupling of diverse cortical and subcortical regions in patients with CD.

Deficits in the sensory, motor, and cognitive processes have been observed in people with the sectioned corpus callosum [20, 21], which is the largest commissural fiber bundle connecting the left and right cerebral hemispheres. This result highlighted the importance of interhemispheric coordination in human behavior. CD is generally characterized by lateralized involuntary movements, which are chronically stereotyped in a predominant direction for each individual. Such movements are likely associated with imbalance between the left and right hemispheres. These data indicated that interhemispheric interaction deficits can play a key role in the neuropathological mechanism of CD. However, previous studies did not focus on possible interhemispheric FC. Therefore, interhemispheric FC in patients with CD should be further investigated. A previously validated method based on rs-fMRI data, voxel-mirrored homotopic connectivity (VMHC), allows the examination of the rs-FC between each voxel in one hemisphere and its mirrored voxel in the opposite hemisphere [22]. Moreover, in previous rs-fMRI investigations, FC analysis was mainly investigated using either seed-based analysis or ICA. The two approaches are useful in evaluating connectivity patterns for specific brain regions or distinct components of brain network but do not reflect the whole connectivity pattern in all brain elements. By contrast, a newly proposed approach called degree centrality focuses on global brain FC and measures FCs between one region and the rest of the brain [23]. This result reflects the functional importance of individual nodes within the whole brain. This graph-based method does not require prior knowledge of the target brain regions. Thus, degree centrality is highly suitable in studying diseases with unclear pathological mechanisms. This method can provide varying brain function information from VMHC. The VMHC and degree centrality methods are reliable and reproducible rs-fMRI metrics [24] and have been successfully used in neuropsychiatric disorders [25, 26].

A combination analysis of complementary analytical approaches may be an effective strategy to achieve a comprehensive description of neural alterations in whole-brain functional network, thereby contributing for the further exploration of new intervention strategies. The present study is the first to perform the combination of the VMHC and degree centrality methods on the same patients to investigate the homotopic FC and local features of whole-brain FC during resting state. Given that structural and functional changes are mainly distributed in the basal ganglia and brain regions corresponding to SMN [13, 17–19], we hypothesized that patients with CD would manifest abnormality in the interhemispheric connectivity and the connectivity strength within the whole brain in certain areas. We expected the brain regions within the SMN and the basal ganglia to be particularly affected. Correlations between altered FCs and clinical variables were also investigated in the patients.

## 2. Materials and Methods

### 2.1. Participants

Twenty-one outpatients with CD were recruited from the Department of Neurology, the First Affiliated Hospital of Guangxi Medical University, China. Among these patients, two cases were excluded because of excessive head movement. All eligible patients met the following inclusion criteria: (1) diagnosis of primary CD with a predominantly rotational abnormality and absence of any evident dystonic symptom in other body parts other than the cervical region; (2) absence of any structural abnormalities, as confirmed by conventional MRI examination; (3) no botulinum toxin injections and neurotropic medication use within 3 months to minimize potential interference effects; (4) right-handedness (Edinburgh Handedness Inventory); and (5) no history of neurological and psychiatric disorders. A total of 21 healthy controls were recruited from the community. The controls were right-handed and group-matched in terms of age, sex, and education level with the patients. Healthy controls had no history of severe medical or neuropsychiatric diseases and family history of neurological or psychiatric disorders in their first-degree relatives.

Information on general demographic characteristics, including sex, age, and years of education, was collected from all participants. Tsui rating scale [27] was used to measure the dystonia severity of the patients. The study was approved by the ethics committee of the First Affiliated Hospital of Guangxi Medical University. All participants were informed about the study procedures and signed a written informed consent before any procedure was initiated.

### 2.2. Data Acquisition

Image data were acquired by using a Siemens (Trio) 3T scanner (Siemens, Erlangen, Germany). Head holders and earplugs were used by all participants to minimize head movement and reduce scanner noise, respectively. During scanning, participants were instructed to lie motionless with their eyes closed without falling asleep. Resting-state functional images were acquired with an echoplanar imaging sequence by using the following parameters: repetition time/echo time = 2000/30 ms, slices = 30, matrix = 64 × 64, flip angle = 90°, field of view = 24 cm, thickness = 4 mm, gap = 0.4 mm, and 250 volumes (500 s). Routine anatomical T1- and T2-weighted images were obtained for spatial localization to rule out intracranial lesions.

### 2.3. Data Preprocessing

Functional image data were preprocessed in MATLAB (https://www.mathworks.com) by using the Data Processing & Analysis for Brain Imaging (DPABI) software. The first 10 volumes were removed to ensure a steady-state condition. After slice timing and head motion, the data of two patients were excluded because of the excessive head motion (exceeding 2° of maximal rotation and/or 2 mm maximum displacement in *x*, *y*, or *z*). Then, the generated images were spatially normalized to the standard Montreal Neurological Institute EPI template in the SPM8 (https://www.fil.ion.ucl.ac.uk/spm) and resampled to 3 mm × 3 mm × 3 mm voxels with the normalization parameters. The normalized and modulated volumes were smoothed with a 4 mm full width at half maximum (FWHM) Gaussian kernel. Finally, the voxels were further temporally bandpass filtered (0.01–0.08 Hz) to reduce the effect of low-frequency drifts and high-frequency physiological noise. Linear trends were removed within each time series. Linear regression was also applied to remove the several sources of spurious covariates and their temporal derivatives, including 24 rigid body head motion parameters obtained by head motion correction, the signal from a region centered in the white matter, and the signal from cerebrospinal fluid. Global signal was not regressed out as suggested in a previous study because it may reflect important neuronal components in the resting-state FC data [28].

### 2.4. VMHC Analysis

The software REST (http://resting-fmri.sourceforge.net) was used to process VMHC. Pearson correlations between each voxel and its mirrored voxel in the opposite hemisphere were computed for each participant. Correlation values were then Fisher *z*-transformed to improve normality.

### 2.5. Degree Centrality Calculation

Weighted degree centrality measures were calculated using REST [29]. The degree centrality value was calculated for each voxel by first extracting its time series. Pearson's correlation coefficients (*r*) were computed between the time series of the voxel and those of all other voxels' time series in a gray matter mask, in which gray matter voxels with *P* > 0.2 were classified. Thus, a whole-brain FC matrix was obtained for each participant. Subsequently, individual correlation matrices were converted into *Z*-scores by using Fisher's *r*-to-*z* transformation to improve normality. The weighted degree centrality strength of a voxel was further calculated as the sum of the connections (*Z*-values) between a given voxel and all other voxels to characterize regional central roles.

### 2.6. Statistical Analysis

The demographic and clinical data were compared with independent two-sample *t*-tests or a Chi-squared test (*P* < 0.05) by SPSS (version 23.0). Two-sample *t*-tests were conducted to compare the differences in VMHC and degree centrality between two groups with the software REST. The resting-state FC can be influenced by micromotions from volume to volume. Thus, the framewise displacement (FD) value for each participant was calculated as the covariate of no interest in the group level comparisons of the VMHC and degree centrality results. Gender, age, and years of education were also regarded as covariates of no interest. Using Gaussian random field (GRF) theory, the significance level was set at *P* < 0.05 with correction for multiple comparisons (voxel significance: *P* < 0.001; cluster significance: *P* < 0.05).

After assessing data normality, the relationship between abnormal VMHC/degree centrality values in regions with significant group differences and clinical variables (illness duration or symptom severity) in the patients was identified using the multiple linear regression (MLR) method. Gender, age, years of education, and FD value were taken as the covariates of no interest in the correlation analyses. The statistical results were set at *P* < 0.05.

## 3. Result

### 3.1. Demographics and Clinical Characteristics

Data from two patients with CD were excluded for further analysis because of excessive head movement. The final list of participants included 19 patients with CD and 21 healthy controls (Table 1). The patients and controls did not exhibit significant group differences in age (patients, 38.74 ± 10.71 years old; controls, 39.62 ± 6.62 years old; *t*-test, *P* = 0.220) and sex (10 female patients and 15 female controls; a Chi-squared test, *P* = 0.759). The mean disease duration of patients was 24.29 ± 31.26 months, and the mean severity score was 16.32 ± 4.45, as determined using Tsui rating scale (0–25). The difference in the FD values between the two groups was not significant (patients, 0.02 ± 0.02 mm; controls, 0.03 ± 0.02 mm; *t*-test, *P* = 0.513). Seventeen participants with CD (89.50%) were observed to transiently obtain at least partial benefit from light touching of lower face, chin, and posterior neck and leaning against the wall. The phenomenon is a sensory trick that is also called as “geste antagoniste” or “alleviating maneuvers” [30]. In the GRF correction for multiple comparisons, the spatial smoothness and RESEL count are as follows: VMHC:FWHMx = 10.718056 mm, FWHMy = 12.177730 mm, FWHMz = 10.796337 mm, and RESELS = 33.086441; degree centrality: FWHMx = 8.280609 mm, FWHMy = 9.091060 mm, FWHMz = 8.906956 mm, and RESELS = 24.833754.

### 3.2. VMHC: Group Differences between Patients with CD and Healthy Controls

**Figure 1:**
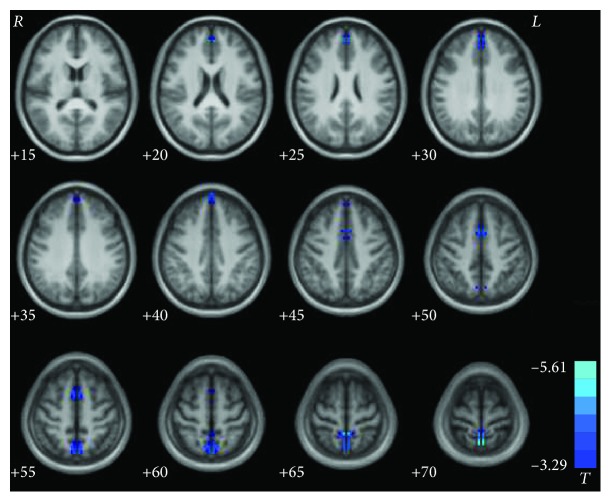
Statistical maps showing VMHC differences between patients and healthy controls. Blue denotes lower VMHC values, and the color bar indicates *T* values from *t*-tests between groups (*P* < 0.05, GRF corrected). VMHC = voxel-mirrored homotopic connectivity.

Figure 1 and Table 2 show significant group differences in VMHC between patients and healthy controls. Compared with the controls, the patients with CD exhibited decreased VMHC in the supplementary motor area (SMA), precuneus (PCu)/postcentral gyrus, and superior medial prefrontal cortex (MPFC). No regions showed increased VMHC in the patient group relative to the control group.

### 3.3. Degree Centrality: Patients with CD versus Healthy Controls

**Figure 2:**
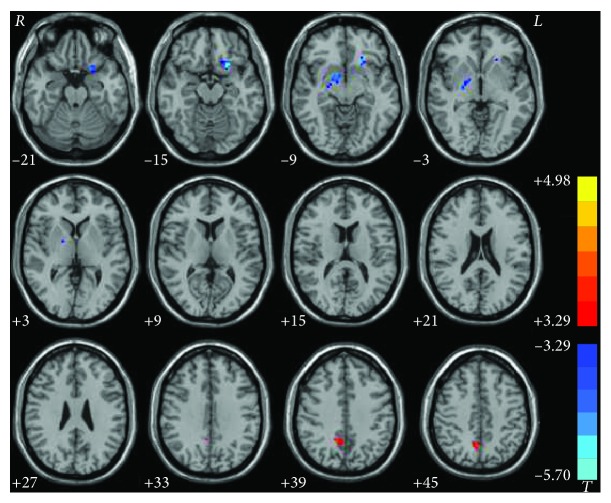
Statistical maps showing degree centrality differences between patients and controls. Red and blue denote higher and lower degree centrality values, respectively, in the patients compared to the controls. The color bars indicate the *T* values of the group analysis (*P* < 0.05, GRF corrected).

**Figure 3:**
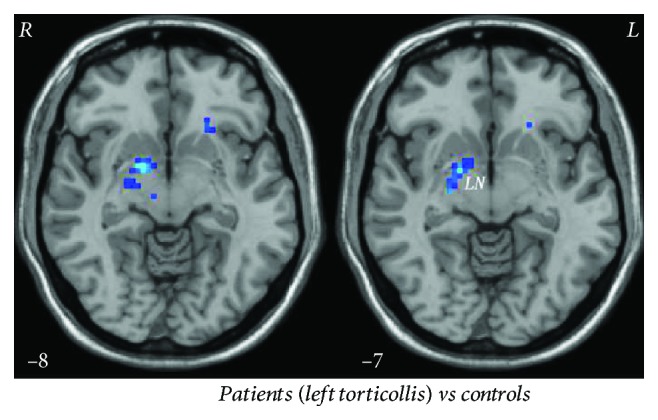
Significant degree centrality differences in the right lentiform nucleus between patients with the left torticollis and controls. Blue denotes lower degree centrality values.

The CD group demonstrated a significantly increased degree centrality in the right PCu and decreased degree centrality in the right lentiform nucleus and left ventral MPFC compared with healthy controls (Figure 2 and Table 3). Additionally, subgroup analysis exhibited a degree centrality decrease in the right lentiform nucleus for the left torticollis group (*x*, *y*, *z* = 15, 0, -9; voxel = 50; *t* = −5.6970), and no significant difference in the left lentiform nucleus for the right torticollis group compared to that of the control group (Figure 3).

### 3.4. Correlation Analysis

**Figure 4:**
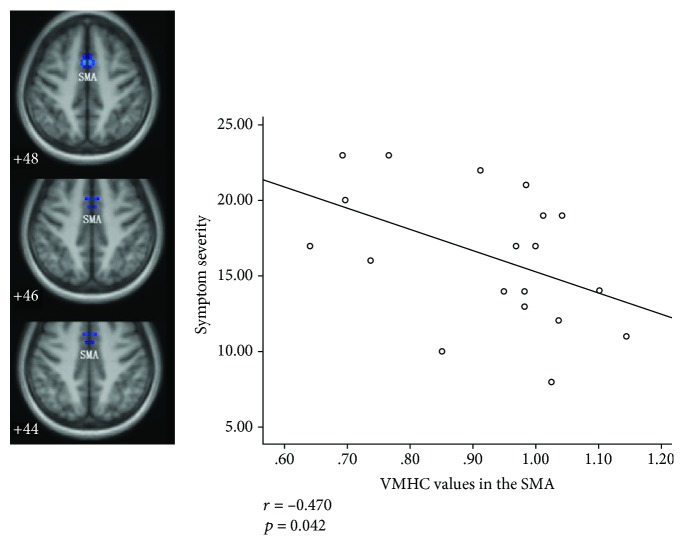
Correlation between the VMHC values in the SMA and symptom severity in the patient group. VMHC = voxel-mirrored homotopic connectivity; SMA = supplementary motor area.

**Figure 5:**
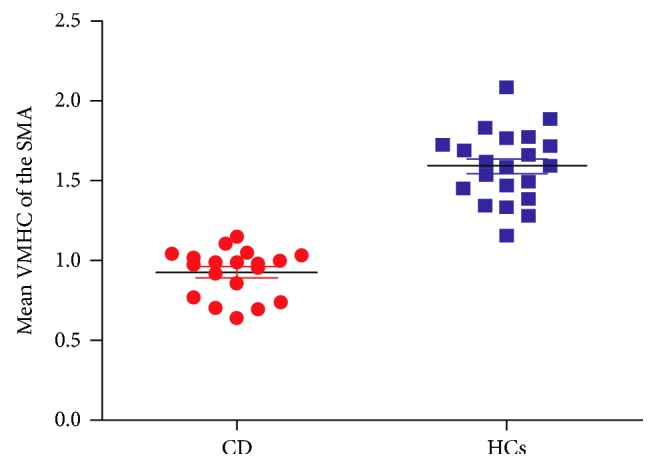
The scatterplot representing group comparisons of the mean VMHC values in the SMA. SMA = supplementary motor area; VMHC = voxel-mirrored homotopic connectivity; CD = cervical dystonia; HCs = healthy controls.

MLR analyses in the patient group showed significantly a negative correlation between symptom severity and VMHC values in the SMA (*r* = −0.470, *P* = 0.042; Figure 4).  Figure 5 shows the mean VMHC values in the SMA for subjects in both CD and HC groups.

## 4. Discussion

In the present study, the VMHC and degree centrality methods were used in combination to explore the possible alterations of the whole-brain FC pattern in patients with CD. Compared with healthy controls, patients with CD exhibited significantly decreased VMHC values in the PCu/postcentral gyrus, SMA, and superior MPFC. Increased degree centrality in the right PCu and decreased degree centrality in the right lentiform nucleus and left ventral MPFC were also found in the patients. Furthermore, the VMHC values in the SMA were significantly correlated with dystonia severity in the patients.

Traditionally, dystonia is regarded as a pure motor disorder derived predominantly from basal ganglia dysfunction on the basis of the evidence from pathophysiology, neurosurgery, animal model, and neuroimaging studies [12]. Decreased degree centrality values were found in the right lentiform nucleus in the patients, supporting an important role of the basal ganglia in the development of dystonia. Further subgroup analysis (12 patients with left torticollis and 7 patients with right torticollis) showed decreased centrality degree in the right lentiform nucleus for the patients with left torticollis and no significant change in the left lentiform nucleus for the patients with right torticollis. A high-resolution MRI study found a significant gray matter increase in the right globus pallidus internus for CD patients with right torticollis [31]. These findings reflect distinctive asymmetrical changes in the basal ganglia in CD and its uncertain relationship with lateralized symptoms. The uncertainty accommodates the complex pattern of muscle involvement in CD, which is characterized by involuntary cocontractions of agonist and antagonist muscles. Moreover, these neural alterations might be a pathophysiological origin and compensatory adjustments for abnormal head postures.

Many functional imaging studies of dystonia have reported the occurrence of dysfunction across cortical and subcortical structures, including several motor and sensory regions [10–12]. Thus, dystonia tends to be a circuit or network disorder. This disease is particularly relevant to the cortico-striato-pallido-thalamo-cortical circuit, which comprises the SMA and postcentral gyrus beyond the basal ganglia [32]. Our findings showing decreased VMHC in the three brain areas were in accordance with the concept that the motor circuit is closely involved in the pathophysiology of primary dystonia [33] and its dysfunction may arise from any nodes or aberrant communication. However, the definite mechanism of circuit dysfunction responsible for the generation of dystonia is unclear.

Recently, the focus on pathophysiology mechanism in dystonia has progressively switched to sensorimotor integration, where sensory information is integrated into the appropriate motor output [34]. Converging evidence indicates that primary dystonia may be associated with sensorimotor integration dysfunction in the motor circuit [35, 36]. There has been general cognition that the SMA is a high-level motor region that involves the preparation and control of movements and complex movements planning, such as posture regulation and voluntary action [37–39]. According to the models of sensorimotor integration, proper sensorimotor integration indicates the coordination of high-level and low-level nodes, and SMA participates in the integration as a high-level node [34, 38]. In the models, SMA modulates the signals from low-level nodes, such as the basal ganglia, thalamus, and cerebellum, and then projects back to the primary motor cortex to calibrate motor execution commands collaboratively with other high-level nodes (i.e., premotor cortex and primary somatosensory cortex). In addition to its involvement in the sensorimotor integration as a crucial node, SMA also acts as a neural correlate of motor inhibition in patients with focal dystonia [40, 41]. These findings indicated that SMA may be closely related to the pathophysiology of CD. Abnormalities in the SMA have been frequently found in the functional and structural MRI studies of dystonia [42–44]. An important result of our study is decreased VMHC in the SMA. The VMHC values in the SMA were inversely correlated with the severity of dystonia in the patients. The negative correlation was in favor of the manifestation of decreased VMHC in the SMA, indicating that the low interhemispheric interaction of this region was related to the severe symptom. Thus, the alteration in SMA function may be applied as an estimation indicator for symptom severity in CD. In early intervention studies, the globus pallidus interna and subthalamic nucleus of the motor circuit have been well-recognized and widely used as the deep brain stimulation targets for effective modulation of dystonia [12]. Repetitive transcranial magnetic stimulation (rTMS) has emerged as a promising adjunctive therapeutic modality in movement disorders. Multiple trials by using low-frequency rTMS over the premotor cortex, motor cortex, and SMA have shown symptom modulation in focal dystonia, including focal hand dystonia [45], writer's cramp [46], and CD [10, 47]. SMA has considerable potential as an alternative target for the further exploration of intervention strategies.

A total of 83% (17/19) patients with CD achieved considerable alleviation by light touches on their lower face, chin, and posterior neck and by leaning against the wall. The phenomenon is called sensory tricks, which is a characteristic and diagnostic feature of primary dystonia that can be observed in approximately 70% of the patients [48]. Although the mechanism is not well understood, there has been recognition for the involvement of a variety of sensorimotor processes in mediating this phenomenon [35, 48]. For example, a PET study showed a reduced activation of the SMA and primary sensorimotor cortex in the application of the sensory trick; thus, the involvement of sensorimotor integration is considered [14]. The presence of sensory tricks suggested that the sensory system is involved in the pathophysiology of dystonia at the sensorimotor level. Previous neuroimaging studies revealed functional and microstructural changes in the primary somatosensory cortex (S1) in patients with CD [15, 19, 49, 50]. The S1 is located on the postcentral gyrus, which is a higher-order sensory receptive area that receives sensory afferents from the bulk of the thalamocortical projection and responsible for somatosensory processing. Generally, improved efficiency can be achieved if the two hemispheres interact when performing all processes compared with one hemisphere alone. Meanwhile, sensory/motor regions have stronger homotopic resting-state FC than hemispherically specialized frontal and parietal association cortices [51]. Therefore, decreased interhemispheric interaction in the bilateral postcentral gyrus indicates insufficient interhemispheric cooperation of sensory processing, thereby contributing to sensorimotor impairment in the present study. Changes in the postcentral gyrus showing decreased VMHC further supported the hypothesis that dystonia is a sensorimotor integration disorder, and the sensory cortex is the key structure of the sensorimotor circuits.

Additionally, brain regions identified from the two measures include the PCu, which manifested decreased VMHC and increased degree centrality values in the right hemisphere. Neurophysiological and functional neuroimaging studies in primates and humans have consistently confirmed that PCu is an associative cortical region exhibiting widespread connectivity with both cortical and subcortical regions to integrate both external and self-generated information to enroll in a wide range of behavioral functions [52–54]. PCu is involved in self-related processing, conscious information processing, episodic memory, and visuospatial processing. The widespread connectivity of PCu allows it to serve functions not limited to the aspects mentioned above. PCu combines information from somatosensory, visual, and other multiple pieces of sensory input [53, 55], thereby contributing to sensorimotor integration and participating in higher-level cognitive functions related to formation of movement intentions or early plans for movement [56]. A FC investigation of this region in humans and macaque monkeys revealed that the anterior PCu functionally connects with the superior parietal cortex, paracentral lobule, and motor cortex, thereby suggesting that it is a sensorimotor region [57]. Considering the evidence mentioned above, PCu may play a role in sensorimotor integration. Reduced FC is thought to reflect impaired function of the network, and increased FC is generally interpreted as compensatory reallocation or dedifferentiation [58]. In previous findings, CD was linked to multiple cortices but rarely to the PCu [15, 17–19]. Thus, our findings extended previous understandings that the PCu may also play a role in the pathophysiology underlying CD. Our finding on the PCu reinforced the pivotal role of sensorimotor integration in the development of dystonia.

The FC measures of the rs-fMRI data have identified well a set of spatially coherent patterns in the human brain, namely, resting-state networks. Among these networks, SMN comprises distributed but functionally related brain regions, including the precentral gyrus, postcentral gyrus, premotor cortex, SMA, and secondary somatosensory cortex [59]. The network primarily concerned with motor planning, motor execution, and sensory processing [16, 60] is related to sensorimotor integration. PCu is also involved in SMN [61]. Together with previous fMRI studies using network analysis [17], impaired function in the postcentral gyrus and SMA, as well as PCu, in our study indicated the loss of connectivity in the SMN in patients with CD compared with healthy controls. These findings provided strong support for the importance of the SMN in the neurophysiology of this movement disorder.

It is interesting that the present study demonstrated significantly decreased VMHC and degree centrality values in the medial prefrontal cortex (MPFC) in patients with CD. MPFC is widely connected to emotional processing areas and limbic areas; thus, it has been implicated in self-referential processing and emotional regulation [62]. The functional and structural differences in the MPFC are related to the modulation of emotional behavior in major depressive disorder [63]. This result suggested that MPFC is important in the emotion-related mechanisms. In addition to movement disorder, nonmotor features, particularly mood disorder, are present in many patients with primary dystonia. Studies have reported a high comorbid incidence of depression and anxiety in patients with dystonia, thereby indicating that these features are the independent manifestation of dystonia [64]. Hence, decreased interhemispheric and whole-brain FC in the MPFC may be involved in nonmotor symptoms, such as depression and anxiety. However, MPFC not only projects to the limbic regions but also connects to the motor system. MPFC contains Brodmann area 10, which is classified as a supramodal prefrontal, in Benson's hierarchical schema [65]. The area is at the top of information processing stream, and information is represented at the most abstract level. Information has a downward trend from the supramodal prefrontal areas to the primary motor cortex through a series of intermediate premotor areas to convert relatively abstract goals in the MPFC into motor plans in the premotor system and finally into the more concrete representations of motor unit activity in the primary motor cortex [66]. Thus, MPFC plays an important role in motor planning and formation.

PCu and MPFC were altered in the VMHC and degree centrality approaches. This result revealed abnormal interhemispheric interaction and local function deficits in the areas. The common alterations may indicate that these areas are involved in the extension of CD neurobiology beyond the SMA. In general, PCu and MPFC are the critical nodes of the default mode network (DMN) [67]. Brain regions within the DMN are among the most globally connected areas responsible for coordinating information throughout the brain [68]. In the present study, the findings on PCu and MPFC suggested the existence of DMN disconnections in patients with CD. Abnormal DMN integrity has been commonly involved in the pathophysiology of psychiatric disorders, such as major depressive disorder [69]. Abnormal DMN integrity is also reported in movement disorders, such as Parkinson's disease and essential tremor [70, 71]. In addition to nonmotor symptoms, network integrity can be involved in the pathogenesis of motor manifestations associated with CD. PCu and MPFC are associated with motor processes on the basis of the connection to motor-related areas. Therefore, such a motor relationship for the DMN may be due to the fact that the representation of motor components within the DMN is disrupted in CD. However, definite consensus is lacking for the specific mechanism of the DMN regarding the modulation of a wide variety of brain functions.

Many researchers claim that the cerebellum and brain stem are involved in the pathophysiology of dystonia, particularly the cerebellum [72, 73]. Therefore, no significant changes in the cerebellar regions and brain stem were unexpected in the present study. There are a few possible interpretations for the absence. First, the cerebellum interacts with the cerebral cortices and basal ganglia through the cerebellar-thalamo-striatal and cerebellar-thalamo-cortical projection pathways [73]. Thus, the cerebellum may act as its roles through the two pathways, although the changes in the cerebellum and brain stem are not predominant in the present study. Second, the differences in cohort characteristics (e.g., sample size and heterogeneity of subjects) and analysis methods may exist. The changes in the cerebellum and brain stem may not be sufficiently predominant to be detected by the VMHC and degree centrality method. Finally, we used a relatively restrict corrected method (GRF corrected at *P* < 0.05 with voxel significance of *P* < 0.001 and cluster significance of *P* < 0.05). When we loosen our *P* level at an uncorrected *P* < 0.001, the cerebellum and brain stem also showed differences between the two groups (data are not shown). Hence, the cerebellum and brain stem may contribute to the pathophysiology of dystonia to some extent.

Except for the small sample size, several limitations in the present study should be acknowledged. Similar to most functional MRI studies, the present study is cross-sectional. Therefore, this work is limited in its discrimination of the causes from the effects of dystonia. The future longitudinal assessments of the pretreatment to posttreatment changes or illness duration are necessary to elucidate this issue. Previous studies also reported that the focal dystonia tends to show comorbidity with anxiety and depressive symptoms. Thus, the assessment of possible effects of the cooccurring psychological symptoms on the functional alterations in patients with CD is a relevant subject for future investigations.

## 5. Conclusions

This study is the first to adopt both the VMHC and degree centrality methods to investigate differences in resting-state brain function between patients with CD and healthy controls. In addition to the basal ganglia (the right lentiform nucleus), the main statistically significant cores entered the SMA and the postcentral gyrus within the SMN, as well as the PCu and MPFC within the DMN. Local abnormalities and interhemispheric interaction deficits in these brain areas may be the key characteristics in the pathogenesis mechanism of CD. The present results confirmed the previous results that support the role of sensorimotor integration in the development of CD. Meanwhile, from a network perspective, our findings highlighted the significance of SMN and DMN in the pathophysiology of CD.

## Figures and Tables

**Table 1:** Clinical characteristics of 19 CD patients and 21 controls.

Patient no.	Gender (M/F)	Handedness	Age (years)	Direction of torticollis	Duration (months)	Tsui score
Sub-01	F	R	32	Right	26	19
Sub-02	F	R	55	Left	18	17
Sub-03	M	R	51	Right	39	16
Sub-04	M	R	20	Right	6	13
Sub-05	F	R	30	Left	1	12
Sub-06	M	R	31	Right	4	22
Sub-07	M	R	41	Left	60	17
Sub-08	F	R	39	Right	7	23
Sub-09	M	R	24	Left	6	21
Sub-10	M	R	50	Left	24	14
Sub-11	M	R	40	Left	6	19
Sub-12	F	R	41	Left	1.5	17
Sub-13	M	R	39	Left	24	8
Sub-14	F	R	58	Right	27	14
Sub-15	F	R	39	Left	24	14
Sub-16	F	R	47	Left	132	23
Sub-17	F	R	45	Right	51	10
Sub-18	F	R	25	Left	4	20
Sub-19	M	R	29	Left	1	11
Mean	9/10 (M/F)	R	38.74 ± 10.71	12/7 (L/R)	24.29 ± 1.26	16.32 ± 4.45
Controls	6/15 (M/F)	R	39.62 ± 6.62			

**Table 2:** Significant group differences in VMHC.

Cluster location	Peak (MNI)			Number of voxels	*T* value
	*x*	*y*	*z*		
Patients < controls					
Superior MPFC	±3	57	42	62	-4.5031
SMA	±3	9	48	58	-4.1644
Precuneus/postcentral gyrus	±3	-60	69	140	-5.6142

MNI = Montreal Neurological Institute; VMHC = voxel-mirrored homotopic connectivity; MPFC = medial prefrontal cortex; SMA = supplementary motor area.

**Table 3:** Difference of degree centrality in patients with CD and control subjects.

Cluster location	Peak (MNI)			Number of voxels	*T* value
	*x*	*y*	*z*		
Patients > controls					
Right precuneus	12	-54	39	39	3.8482
Patients < controls					
Left ventral MPFC	-21	18	-15	53	-5.5465
Right lentiform nucleus	15	0	-9	39	-4.7977

CD = cervical dystonia; MPFC = medial prefrontal cortex; MNI = Montreal Neurological Institute.

## Data Availability

The data that support the findings of this study are available from the corresponding authors upon request.
